# Total Polyphenol and Flavonoid Content and Antioxidant Capacity of Some Varieties of *Persea americana* Peels Consumed in Cameroon

**DOI:** 10.1155/2021/8882594

**Published:** 2021-04-21

**Authors:** Ruth Edwige Kemadjou Dibacto, Boris Ronald Tonou Tchuente, Maxwell Wandji Nguedjo, Yves Martial Tongue Tientcheu, Emilienne Carine Nyobe, Ferdinand Lanvin Ebouel Edoun, Melanie Flore Godam Kamini, Romelle Feumba Dibanda, Gabriel Nama Medoua

**Affiliations:** ^1^Centre for Food and Nutrition Research, Institute of Medical Research and Medicinal Plants Studies, Ministry of Scientific Research and Innovation, Yaounde, Cameroon; ^2^Department of Biochemistry, University of Yaounde1, Yaounde, Cameroon; ^3^Department of Plant Biology and Physiology, University of Yaounde1, Yaounde, Cameroon; ^4^Department of Biochemistry and Molecular Biology, Faculty of Sciences, University of Buea, Buea, Cameroon

## Abstract

Fruit peels are increasingly being used as functional foods nowadays. Peelings of twelve varieties of *Persea americana* fruits consumed in Cameroon were investigated for their phenolic compounds (polyphenols and flavonoids) using three solvents systems, water, ethanol: water (50 : 50 *v*/*v*), and ethanol, and antioxidant activity using total antioxidant capacity (TAC), ferric reducing antioxidant power (FRAP), and 1,1-diphenyl-2-picryl hydrazyl (DPPH) radical scavenging methods. Total polyphenol, flavonoids, and antioxidant potential of the peels significantly varied with *P. americana* variety and also with the extraction solvents in the order ethanol > ethanol: water > water. Total phenolic content varied from 2407 (Fuerte florid) to 673 (Semil) mg GAE/g DM, respectively, while flavonoids varied from 986 to 119 mg QE/g DM for Fuerte florid and Hickson varieties, respectively. TAC, respectively, varied between 132.87 and 126.85 mg AAE/g DM with Hass and Semil varieties, respectively. The highest DPPH scavenging capacity was recorded for the ethanolic extract with Lula (86.33%) and the least for the aqueous extract with the Semil (30.11%) variety. With FRAP, the highest capacity was obtained with hydroethanolic extract of Fuerte florid (0.43 mg AAE/g DM) and the least for aqueous extract with the Semil (0.269 mg AAE/g DM) variety. In conclusion, varieties of avocado peels are a good source of antioxidants. Solvent extraction significantly affected the concentration of bioactive compounds but not the potency of the antioxidants. A weakly positive correlation but not significant between the quantity of polyphenol, flavonoid, and antioxidant capacity of avocado peelings was obtained in this study.

## 1. Introduction

In living organisms, cellular respiration generates reactive oxygen species which are very unstable and react rapidly with other substances, including DNA, membrane lipids, and proteins, resulting in health disorders such as diabetes mellitus, hypertension, cancer, neurodegenerative, and other disorders [1]. The consumption of antioxidant-rich food products has been positively related to a reduction in the risk of developing these chronic diseases [2]. Antioxidants are molecules that prevent or slow down oxidation by neutralizing free radicals [3]. Nowadays, natural antioxidant ingredients from plants food are being preferred by the food industry [4] compared to synthetic antioxidants such as butylated hydroxylanisole which have been reported to be toxic at high concentrations [5]. Several studies reported that much of the total antioxidant activity is related to the phenolic content of the food products [6–8]. Fruits, vegetables, and many other plants have been reported to be rich in phenolic compounds [6, 9, 10].

Avocado (*Persea americana*) belongs to the Lauraceae family. It is native to Central America and Mexico and cultivated in almost all tropical and subtropical regions of the world [11, 12]. Due to their nutritional characteristics [13], all the varieties of avocado (Lula, Nabal, Hickson, Booth 8, Semil, Booth 7, Taylor, Collinson, Anaheim, Hass, Zutano, Fuerte florid, and Shepard) are widely consumed worldwide. In fact, the pulp of avocado is very rich in unsaturated fatty acids, vitamin B, C, and E, and several nutrients such as potassium and dietary fibers [14–16]. Several studies on avocado highlight the use of its oil for medicinal purposes [17].

Avocado is one of such fruits whose processing generates a large amount of waste. When the pulp is used, the seeds and peels are discarded as wastes, constituting environmental challenges. The aqueous extracts of avocado seeds showed good antioxidant properties useful against lipid peroxidation [10]. Some studies were carried out on the phenolic content and the antioxidant capacity of peels of three varieties of avocado, namely, Hass, Fuerte, and Shepard [7, 9], and they demonstrated that avocado peels could be consumed as functional ingredients. Its high content of nutrients and bioactive phytochemicals such as antioxidants makes it a “superfood” [18].

In Cameroon, there are many varieties of avocado available during the whole year and widely consumed all over the country. They are densely produced in the West Region of Cameroon which appears as the main production basin. Once consumed, stones and peels which are considered as wastes are thrown into garbage. Until today, to our knowledge, research studies carried out in sub-Saharan Africa and particularly in Cameroon, in view of the valorisation of these wastes, were limited to avocado stones. Studies performed by Azantsa [19] revealed the toxic effect of avocado stones. However, in other continents such as Europe and America, there are studies which demonstrated the presence of bioactive compounds in avocado peels. The authors highlighted a great variation of bioactive compounds of avocado peels according to the regions and the climatic conditions [20]. Hence, it therefore appears interesting to assess the levels of bioactive compounds found in avocado peels which are considered as wastes in Cameroon in order to increase their added value. It is in view of this output that the present study was designed. The objective was to investigate the phenolic content and the antioxidant properties of avocado peels which can be used as functional ingredients in the formulation of antioxidants-rich food products useful in the management of chronic diseases.

## 2. Materials and Methods

### 2.1. Chemical Reagents

The following chemicals were used in this study: Folin-Ciocalteu [(HO) 3C_6_H_2_CO_2_, H_2_O; Merck], sodium carbonate (Na_2_CO_3_, Merck), gallic acid (C_7_H_6_O_5_; Merck), aluminum chloride (AlCl_3_, Merck), potassium acetate (CH_3_COOK; Merck), quercetin (C_15_H_10_O_7_, 2H_2_O, Merck), ethanol (C_2_H_5_OH 99%, Merck), hydrochloric acid (HCl, 12 N), iron chloride (FeCl_3_; Merck), ascorbic acid (C_6_H_8_O_6_; Merck), sulfuric acid (H_2_SO_4_; 98%), sodium dihydrogen phosphate (NaH_2_PO_4_, Merck), dibasic hydrogen phosphate (Na_2_HPO_4_, Merck), ammonium molybdate (H_24_Mo_7_N_6_O_24_, Merck), potassium ferricyanide [K_3_Fe (CN)_6_, Merck], and trichloroacetic acid (CCl_3_CO_2_H, Merck). All chemicals used were obtained from Sigma Aldrich.

### 2.2. Collection, Identification, and Preparation of Plant Material

Mature but unripe fruits were harvested from avocado trees in the yard of the Institute of Agricultural Research for Development (IRAD) (Foumbot, West region, Cameroon) on 9–13 of August 2019. Five fruits were harvested per variety, and varieties collected were Lula, Nabal, Hickson, Booth 8, Semil, Booth 7, Taylor, Collinson, Anaheim, Hass, Zutano, and Fuerte florid (most consumed varieties identified by IRAD). Fruits were allowed to ripen at room temperature (25–30°C) before being washed with distilled water. Each fruit was divided into four using a stainless knife, and its pulp, seed, and peel were collected separately. And all the pulp adhering to the peel was carefully removed using a stainless spoon. For each variety, peels were gathered, dried at 60°C for 24 h (using an oven), and ground to obtain powders with particle size of 500 *µ*m (with a blinder).

### 2.3. Extract Preparation

Extract of avocado was obtained by weighing 10 g of the powder from each variety into 200 mL extraction solvent at room temperature (25–30°C) and dissolved for 24 hours with intermittent shaking. The mixture was then filtered with Whatman No. 2 paper; the process was repeated twice, and all filtrates were pooled together. The resulting extracts were then chilled for further analysis. The solvents used for extraction were water, ethanol: water (50 : 50 *v*/*v*), and ethanol.

### 2.4. Phytochemical Analysis

#### 2.4.1. Total Polyphenol Content Assessment

Total phenolic content was determined following the method described by Singleton and Rossi [21] with some modifications. Folin-Ciocalteu reagent was used with gallic acid as the standard phenolic compound. About 0.5 mL of an extract was introduced into test tubes followed by 2.5 mL of 10% Folin-Ciocalteu reagent and 2 mL of 7.5% Na_2_CO_3_. The tubes were vigorously homogenized on a shaker, and the mixture was allowed to stand for 30 minutes, and absorbance was read at 765 nm. Total phenol content was expressed as milligram of gallic acid equivalent (GAE) per gram of dry matter extract (mg GAE/g DM).

#### 2.4.2. Total Flavonoid Content Assessment

The colorimetric method described by Aiyegoro and Okoh [22] with aluminum chloride was used to evaluate the total flavonoid content. To 0.2 mL of aluminum chloride (AlCl_3_, 10%) was added 0.2 mL aliquot of an extract followed by the successive addition of 0.2 mL of potassium acetate (CH_3_COOK, 1 M) and 1.12 mL of distilled water. The whole mixture was well homogenized and incubated at room temperature, and the absorbance was read at 415 nm against the reagent blank 30 minutes later. Quercetin (0–1000 *μ*g/mL) served as a standard, and the results were expressed as mg QE/g DM.

### 2.5. Evaluation of Antioxidant Potential

The antioxidant activity was evaluated using methods involving synthetic oxidants such as DPPH, tripyridyl iron complex (FRAP), and phosphotungstinic and molybdic acids.

#### 2.5.1. Trapping of the Radical DPPH (2,2-Diphenyl-1-picrylhydrazyl)

The free radical scavenging ability of the extract against DPPH (1,1-diphenyl-2-picrylhydrazyl) was carried out according to the slightly modified method of Katalinié et al. [23]. To 2.5 ml of the extracts was added 3 ml of ethanol and 0.5 ml of DPPH solution (55 *µ*M). The mixture was shaken and left to stand in the dark at room temperature for 30 min. The absorbance of the resulting mixture was read at 517 nm. The same procedure was repeated using a control sample (DPPH without extracts). Ascorbic acid was used as the standard antioxidant. The scavenging ability of the extracts was calculated as

(1) $$\begin{array}{c} I_{\text{DPPH}}\left(\%\right)=\frac{{\text{Abs}}_{\text{control}}\;-{\text{Abs}}_{\text{essai}}}{{\text{Abs}}_{\text{control}}}\;\times 100\;. \end{array}$$

#### 2.5.2. Determination of Total Antioxidant Capacity

The total antioxidant activity of the extract was evaluated by the formation of phosphomolybdenum complex [24]. To this effect, a 0.2 ml solution of extract was added to 2 mL of reagent solution (0.6 M H_2_SO_4_, 28 mM sodium phosphate, and 4 mM ammonium molybdate). The absorbance was measured at 695 nm after boiling for 60 minutes. Ascorbic acid was used as a standard, and total antioxidant capacity was expressed as milligrams of ascorbic acid equivalent (AAE) per grams of an extract of dry matter (mg AAE/g DM).

#### 2.5.3. Reducing Ferric Capacity (FRAP: Ferric Reducing Antioxidant Power)

The method of Oyaizu [25] was used to assess the reducing power of the fruit peelings extracts. A volume of 1 ml of fruit peelings extract was mixed with 2.5 ml of a 0.2 M sodium phosphate buffer (pH 6.6) and 2.5 ml of potassium ferrocyanide (1%) and incubated in a waterbath at 50°C for 20 min. Then, 2.5 ml of 10% trichloroacetic acid was added to the mixture that was centrifuged at 650*g* for 10 min. The supernatant (2.5 ml) was then mixed with 2.5 ml distilled water and 0.5 ml of 0.1% ferric chloride solution. The intensity of the blue-green color was measured at 700 nm. Ascorbic acid was used as a positive control.

### 2.6. Statistical Analyses

The data were subjected to statistical analysis using Statistical Package for Social Sciences (SPSS) Version 21.0 (SPSS, Inc., IBM Corporation, Chicago, USA). Analysis of variance (ANOVA) was used to determine the means. The Fisher test was used to determine the least significant difference (LSD) of the means. The significance was set at 5% (*P* < 0.05). The experiments were repeated thrice. The strength of the association between these variables was determined using the Neyman–Pearson correlation. Microsoft Excel 2016 (Microsoft Corporation, California, USA) was used to plot the graphs.

## 3. Results

### 3.1. Total Phenolic and Flavonoid Contents of Fruits Peelings

Table 1 provides the presence of total polyphenolic and flavonoid content of fruit peelings. The values of total polyphenol varied in the range from 2407 (Fuerte florid) to 804 (Anaheim) mg GAE/g DM for ethanolic extracts, from 1037 (Fuerte florid) to 703 (Collinson) mg GAE/g DM for hydroethanolic extracts, and from 867 (Fuerte florid) to 673 (Semil) mg GAE/g DM for aqueous extracts. The values flavonoids content ranged from 986.26 (Fuerte florid) to 635 (Anaheim) mg QE/g DM for ethanolic extracts, from 724 (Fuerte florid) to 311 (Hickson) mg QE/g DM for hydroethanolic extracts, and from 427 (Fuerte florid) to 119 (Hickson) mg QE/g DM for aqueous extracts.

### 3.2. Antioxidant Activities of Different Extracts of Avocado Peelings

The antioxidant capacity of natural products is generally evaluated by combining several different in vitro tests to obtain conclusive results. Total antioxidant capacity (TAC), iron reduction (FRAP), and DPPH free radical scavenging methods are commonly used to determine antioxidant potential in vitro.

### 3.3. Evaluation of Total Antioxidant Capacity (TAC)

Table 2 provides the antioxidant capacity of the different extracts of avocado peels as investigated by the total antioxidant capacity method. Values of TAC of hydroethanolic extracts ranged from 132.87 (Hass) to 127.97 (Booth 8) mg EAA/g, meanwhile, that of ethanolic extract ranged from 130.02 (Hass) to 127.32 (Booth 8) mg EAA/g and that of aqueous extracts ranged from 129.47 (Hass) to 126.85 (Booth 8) mg EAA/g based on the dry weight. The highest and lowest total antioxidant capacity was recorded in Hass and Booth 8 varieties, respectively, for all solvent extractions.

### 3.4. DPPH Radical Scavenging Capacity in Avocado Peelings

**Figure 1:**
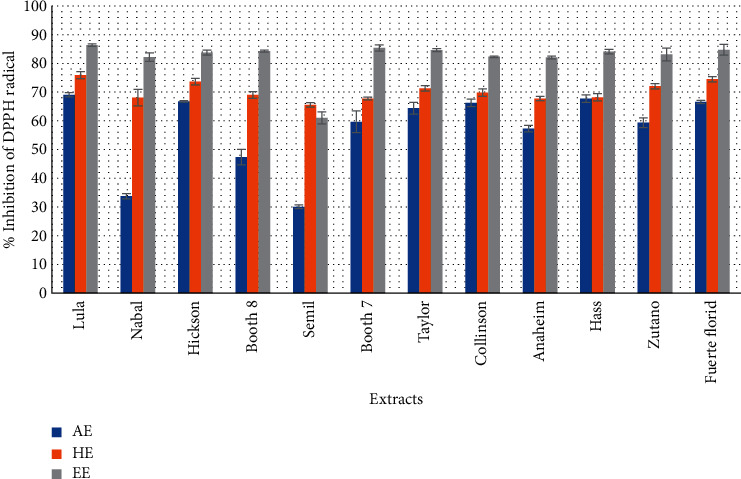
Scavenging capacity of the DPPH radical by aqueous, hydroethanolic, and ethanolic extracts of avocado varieties peelings.

The radical scavenging activity of fruit peelings of *Persea americana* is shown in Figure 1. DPPH scavenging radical scavenging activity in ethanolic, hydroethanolic, and aqueous extracts ranged from 86.33% (Lula) to 61.03% (Semil), from 75.88% (Lula) to 65.55% (Semil), and from 69.07% (Lula) to 30.11% (Semil), respectively; highest activity was observed being in Lula variety and the lowest activity in Semil for all peel extracts.

### 3.5. Iron Reducing Power (FRAP)

**Figure 2:**
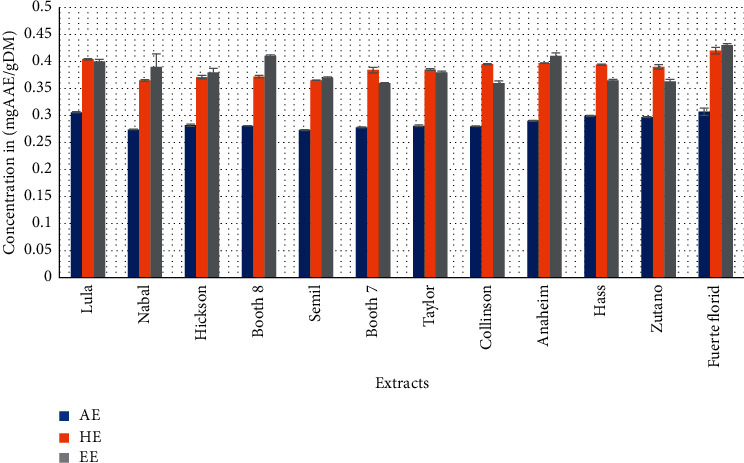
Iron reducing power (FRAP) of aqueous, hydroethanolic, and ethanolic extracts of *Persea americana* fruits peelings.

Figure 2 shows the data on the iron reducing power of different extracts of avocado peels. FRAP values of ethanolic, hydroethanolic, and aqueous extracts range from 0.43 (Fuerte florid) to 0.37 (Semil) mg AAE/g DM, 0.42 (Fuerte florid) to 0.365 (Nabal) mg AAE/g DM, and 0.307 (Fuerte florid) to 0.273 (Semil) mg AAE/g DM, respectively. The highest activity was observed being in the Lula variety (all solvent) and the lowest activity in Semil (ethanolic and aqueous solvents) and Nabal (hydroethanolic) varieties.

### 3.6. Correlation between the Tested Bioactive Compounds Content and Antioxidant Activities of Different Peelings Extracts

Table 3 is a summary of the correlation coefficients of the compounds of interest as a function of the solvents and the antioxidant activities performed. Overall, of the 9 correlation cases performed, all were positive, and only the correlations between FRAP and polyphenols in hydroethanolic and aqueous extracts (*R*^2^ = 0.657; *P*=0.020 and *R*^2^ = 0.577; *P*=0.049, respectively) and FRAP and flavonoids in hydroethanolic (*R*^2^ = 0.705; *P*=0.010) extracts were significant (*P* ≤ 0.05). And for the other tests and solvents not mentioned above, weak but positive and nonsignificant correlations were observed.

## 4. Discussion

This study examined the antioxidant contents of different avocado fruit peelings. It has been determined using the colorimetric method. The presence of these antioxidants implies that avocado peels could have protective and therapeutic implications for humans. Phenols are major contributors to the antioxidant capacity of most plants. The solvent is an important factor in the extraction of bioactive components of plants [26]. The phenolic content of avocado peels was highest in the ethanolic extracts and lowest in the aqueous extracts for all peel varieties. These results are similar to those of Tremocoldi et al. [7] and Romelle et al. [8] who reported a considerable total phenolic content in avocado, mango, apple orange, and banana peels. The total phenolic content of ethanol peel extracts for all varieties was significantly higher than those of hydroethanolic extracts and aqueous extracts. The highest polyphenol content recorded with ethanol as solvent might arise from the fact that phenolic compounds are known to be more soluble in organic solvents [27]. The key role played by the polarity of organic solvents in the solubility of phenolic compounds of plants was highlighted by several reports in the literature [28–30]. Globally, the authors concluded that, compared to other solvents, the organic ones extracted more phenolic compounds independently of the plant material. In another study, it was noticed that polyphenols are often more soluble in organic solvents which are less polar than water [30]. Moreover, the structural diversity of bioactive compounds found in the peel extracts might also explain the difference observed. Indeed, it was demonstrated that the difference in the structure of polyphenols determine their solubility in solvents [28]. The variability of polyphenol and flavonoid contents in these plant species is probably due to the phenolic composition of extracts [31, 32], genotypic factors [33], biotic conditions (species, organ, and physiological stage), and abiotic (edaphic factors) [34]. This may explain the various biological activities, such as anticarcinogenic, anti-inflammatory, and antiatherosclerotic activities, of various parts of avocado. These bioactive compounds (as antioxidants) have protective and therapeutic implications for man.

For centuries, antioxidants from plant extracts or their secondary metabolites have served as phytotherapeutic tools in medicine to protect against various oxidative stress and free radical related diseases. Free radicals include hydroxyl radicals, peroxyl radicals, superoxide radicals, hydrogen peroxide, singlet oxygen, and various lipid peroxides [35]. Considering the variety of oxidation processes and reactions that have been reported [10, 36], a single method cannot be enough to provide a clear idea of the antioxidant potential of a compound. However, a single test cannot judge the antioxidant activity of an extract [37]. In this study, several mechanisms of action (antiradical and reducing activities) were used. Total antioxidant capacity (TAC) analyses showed that all the tested peel extracts displayed the antioxidant activity. The extracts from the twelve varieties of avocado peelings were able to reduce Mo (VI) in Mo (V). Hass variety presented the best total antioxidant capacity in all solvents used and is not the variety with the best phenolic compound content yet. Booth 8 presented the lowest activity but is not the variety with the lowest level of compounds of interest. For all the varieties, the TAC activities were not significantly different (between the solvent system and varieties); we can justify this by the fact that all our samples were collected in the same region (west). The results obtained in the present study showed that TAC of all avocado peel extracts is not dependent on the total phenolic content but rather depends on the configuration and glycosylation of hydroxyl groups present in phenolic compounds in the extracts [38]. More specifically, the screening results revealed the presence of flavonoids that could be the compounds involved in the antiradical and reducing activities of our extracts by electron release. It is widely accepted that high polyphenol content does not necessarily exhibit powerful antioxidant activity [5]. These results are related to the electron-donating capacity of the flavonoids contained in each extract.

For the scavenging activity investigation, the synthetic DPPH radical is widely used. All the extracts of peelings used were able to convert DPPH radicals to more stable species (DPPH-H or DPPH-R). The DPPH method measures the ability of antioxidants present in scavenging the hydrophilic free radicals. Overall, ethanolic extracts of avocado peelings strongly scavenged the DPPH radical with the greatest inhibition with ethanolic extract of Lula and the lowest with aqueous extract of Semil. The reducing power of the extracts measured in this study shows that the antioxidants in the extracts were able to donate electrons and could therefore give up free radicals in biological or food environments by inactivating them. Several authors reported that the reducing power of bioactive compounds (mainly phenolic acids, polyphenols, and flavonoids) extracted from spices, herbs, and medicinal plants was associated with antioxidant activity, particularly free radical scavenging [39]. Antioxidants can be explained as reductants, and inactivation of oxidants by reductants can be described as redox reactions in which one reacting species is reduced at the expense of the oxidation of the other. The reaction between antioxidant compounds and the DPPH radical will cause a reduction in absorbance and decolorization of DPPH from purple to light yellow [40]. One of the main properties of polyphenols is their antiradical activity which is itself attributed to their hydroxyl groups which contribute to their redox properties, electron-donating capacity, singlet antioxygen action, and metal chelating capacity. Indeed, the class of polyphenols has been proven to determine the free radical scavenging activity, the flavones being the most effective [41]. This result was corroborated by Nahak et al. [42] who showed that an antioxidant that is effective in one test is not necessarily effective in another on the one hand. On the other hand, antioxidant activities are more variable in plant species (interspecies) than in the same species (intraspecies), but it can be seen in same species [34].

The FRAP results followed the same trend as the phenol, flavonoids, and DPPH, with ethanolic, followed by hydroethanolic, and finally by aqueous extracts. The presence of reducers in plant extracts causes the reduction of Fe3^+^/ferricyanide complex to the ferrous form. Therefore, Fe2^+^ can be evaluated by measuring and monitoring the increase in the density of the blue color in the reaction medium at 700 nm [43]. Many current publications have indicated that there is a direct relationship between antioxidant activities and the reduction power of components in some plants [44]. Extracts with the highest polyphenols/flavonoids showed the best FRAP activities for all solvent systems. Kaurinovic and Vastag [45] highlighted that flavonoids are strong iron reducers and that the property originated from their catechol moiety. In fact, Folassade et al. [46] demonstrated the chelating activity of free radicals played by polyphenols present in fruits. Moreover, the scavenger effect of flavonoids (FLOH) is attributed to their low redox potential which makes them thermodynamically capable of reducing free radicals (R-) by a transfer of hydrogen atoms from hydroxyl groups. This reaction gives rise to the aroxyl radical (FLO-) and the stabilized radical molecule (RH); the FLO- will subsequently undergo a structural rearrangement allowing the redistribution of the single electron on the aromatic ring and the stabilization of the aroxyl radical [47]. Fuerte florid which showed the highest content of bioactive compounds had the best activity in the test (iron reducing); but neither total antioxidant capacity (Hass) nor scavenging activity (Lula) probably obeys the rule which states that this activity not only depends on the number of bioactive molecule contents but also on the structure of the molecules that form the extract [48]. The other explanation for these results on antioxidant capacity could be due to the synergy of phenolic and nonphenolic compounds. This is why certain extracts with contents more or less low to the others presented remarkable changes in their antioxidant activity. It is widely accepted that high polyphenol content does not necessarily exhibit powerful antioxidant activity [5] and this is what this study shows.

This result obtained with correlation indicates that for these extracts, the results of one test cannot be predicted from the results of the other test. And for the others tests and solvents not mentioned above, weak but positive and nonsignificant correlations were observed. A weak positive correlation with polyphenols content, which is in contrast to many works generally linking both parameters [49], which therefore questions the nature of polyphenols that are present in extract was observed. Other authors have noted a correlation between these methods and more specifically between polyphenol content and free radical scavenging in many spices, vegetables, fruits and beverages, and fruit peels [50]. These positive correlations suggest a contribution of phenolic compounds in free radical scavenging activity. Phenolic and flavonoid molecules are important antioxidant components that are responsible for deactivating free radicals based on their ability to donate hydrogen atoms to free radicals. They also have ideal structural characteristics for free radical scavenging [51]. However, although the correlation between these methods has often been observed in many studies, the magnitude of these correlations may vary from one variety to another since two extracts from the same plant may have antioxidant compounds of different structure and composition that react differently with one or the other method [49]. It is known that phenolic compounds of peels can scavenge free radicals and that the factors such as genetic and environmental conditions (growth season and plant maturity) can cause variations in their values [20]. As it has been seen in this study, with Fuerte florid, the ethanolic extract was first with FRAP, but for TAC, it was rather the hydroethanolic extract that was first with the Hass variety, and for DPPH, the ethanolic extract of the Lula variety was the first which contributed to the low correlation in general between the different antioxidant tests carried out. For the extracts of other plants, their behaviour to give up the proton or electron to express the antioxidant properties is different. Some works have revealed a good correlation between IC50s and polyphenol and flavonoid content, while other studies have not established this correlation [52, 53]. In addition, it is well established that antioxidant activity is positively correlated with polyphenol structure not only on quantity. Generally, polyphenols with a high number of hydroxyl groups have the highest antioxidant activity [54] due to their ability to donate more atoms to stabilize free radicals [55], which may partly explain that the antiradical activity is dependent on the number, position, and nature of substituents on the B and C rings (hydroxyl groups, metalaxyl groups, and glycosylated groups) and the degree of polymerization [56]. Thus, the antioxidant effect is not only dose-dependent but also structure-dependent [57]. The types of polyphenols contained in these extracts are probably responsible for the antioxidant activity. Correlation between DPPH, FRAP, and TAC indicated that phenolic compounds present in different peel extracts exhibit the scavenging ability of DPPH and ferric ion and phosphomolybdate reducing abilities, respectively. The positive correlations between FRAP and other antioxidant assays were in agreement with other studies [58].

## 5. Conclusion

This study showed that varieties of avocado peelings contain considerable amounts of polyphenols and flavonoids, making it a good source of natural antioxidants and concentrations varying from one variety to another. The organic solvent (ethanol) used was more efficient in extracting the bioactive component of fruits of *Persea americana* peels compared to water or the diluted solvents. About the different tests chosen for antioxidant activities on varieties of peels, with the total antioxidant capacity, the best activity was observed with the hydroethanolic extract of Hass; for FRAP, the ethanolic extract of Fuerte florid and, finally for DPPH, the ethanolic extract of Lula showed the best activity. These differences in the antioxidant mechanism can be exploited in the medical field for the management of metabolic diseases (fight against oxidative stress). However, it was found that there was not really a strong correlation between the quantity of bioactive compounds and the antioxidant activities evaluated in this study.

## Figures and Tables

**Table 1:** Total phenolic and flavonoids content of aqueous, hydroethanolic and ethanolic extracts of the different avocado varieties' peelings.

		Avocado varieties' peelings											
		Lula	Nabal	Hickson	Booth 8	Semil	Booth 7	Taylor	Collinson	Anaheim	Hass	Zutano	Fuerte florid
TPC (mg GAE/g DM)	AE	806 ± 8^a^	828 ± 12^a^	700 ± 14^b^	724 ± 12^bc^	673 ± 18^b^	763 ± 13^d^	782 ± 11^d^	751 ± 5^cd^	746 ± 13^cd^	802 ± 8^a^	756 ± 13^d^	867 ± 15^e^
	HE	920 ± 17^a^	885 ± 12^a^	742 ± 11^b^	754 ± 17^bc^	758 ± 8^b^	801 ± 18^c^	733 ± 29^b^	703 ± 10^b^	778 ± 10^b^	813 ± 12^c^	776 ± 13^b^	1037 ± 15^d^
	EE	1430 ± 17^b^	1026 ± 20^c^	1115 ± 26^c^	1088 ± 20^c^	843 ± 28^c^	1513 ± 20^d^	1302 ± 22^b^	887 ± 12^c^	804 ± 13^c^	1836 ± 20^a^	1362 ± 16^b^	2407 ± 21^e^
TFC (mg QE/g DM)	AE	348 ± 2^a^	303 ± 1^b^	119 ± 1^d^	265 ± 1^e^	347 ± 1^a^	137 ± 2^d^	283 ± 3^b^	223 ± 1^e^	162 ± 2^d^	399 ± 0.8c	298 ± 1^b^	427 ± 2^c^
	HE	617 ± 6^c^	406 ± 3^d^	311 ± 6^e^	470 ± 2^b^	429 ± 4^d^	441 ± 0,9^d^	494 ± 3^b^	441 ± 3^b^	367 ± 2^d^	678 ± 2^a^	403 ± 3^b^	724 ± 4^a^
	EE	859 ± 2^b^	836 ± 3^b^	740 ± 2^c^	660 ± 1^d^	504 ± 4^d^	489 ± 7^c^	527 ± 1^d^	499 ± 0,8^c^	535 ± 2^d^	978 ± 8^a^	682 ± 5^d^	986 ± 6^a^

mg GAE/g DM, milligram gallic acid equivalent/gram of dry matter; mg QE/g DM, milligram quercetin equivalent/gram dry matter; TPC, total polyphenol content; FC, flavonoid content; AE, aqueous extract; HE, hydroethanolic extract; EE, ethanolic extract. Any two mean ± SD values that do not carry similar superscript in a line are significantly different (*P* ≤ 0.05).

**Table 2:** Total antioxidant capacity (TAC) of aqueous, hydroethanolic, and ethanolic extracts of avocado varieties' peelings.

	Lula	Nabal	Hickson	Booth 8	Semil	Booth 7	Taylor	Collinson	Anaheim	Hass	Zutano	Fuerte florid
TAC (mg AAE/g DM) AE	128.79 ± 0.004^a^	127.76 ± 0.0256^a^	128.21 ± 0.0641^a^	126.85 ± 0.04^a^	127.34 ± 0.028^a^	129.32 ± 0.018^a^	129.08 ± 0.05^a^	128.56 ± 0.022^a^	129.32 ± 0.032^a^	129.47 ± 0.017^a^	127.21 ± 0.036^a^	127.87 ± 0.083^a^
TAC (mg AAE/g DM) HE	132.41 ± 0.02^a^	129.15 ± 0.0235^a^	129.36 ± 0.068^a^	127.97 ± 0.020^a^	128.47 ± 0.0281^a^	131.08 ± 0.0391^a^	129.26 ± 0.0463^a^	130.34 ± 0.021^a^	131.33 ± 0.033^a^	132.87 ± 0.09^a^	129.53 ± 0.020^a^	130.78 ± 0.026^a^
TAC (mg AAE/g DM) EE	129.63 ± 0.024^a^	127.86 ± 0.092^a^	127.77 ± 0.018^a^	127.32 ± 0.034^a^	127.83 ± 0.02^a^	129.75 ± 0.03^a^	128.26 ± 0.038^a^	129.11 ± 0.03^a^	129.32 ± 0.034^a^	130.02 ± 0.03^a^	127.68 ± 0.057^a^	129.45 ± 0.09^a^

mg AAE/g DM, milligram ascorbic acid equivalent/gram dry matter; AE, aqueous extract; HE, hydroethanolic extract; EE, ethanolic extract; TAC, total antioxidant capacity. Any two mean ± SD values that do not carry similar superscript in a line are significantly different (*P* ≤ 0.05).

**Table 3:** Correlation coefficients and *P* values between the bioactive compounds of different peelings extracts and different methods used to assess the antioxidant activity.

Solvents	Antioxidant tests	Total phenolic	Flavonoids
AE	DPPH	*R* ^2^ = 0.322, *P*=0.308	*R* ^2^ = −0.077, *P*=0.812
	FRAP	*R* ^2^ = 0.577, *P*=0.049	*R* ^2^ = 0.522, *P*=0.082
	TAC	*R* ^2^ = 0.249, *P*=0.435	*R* ^2^ = −0.229, *P*=0.474
HE	DPPH	*R* ^2^ = 0.450, *P*=0.142	*R* ^2^ = 0.340, *P*=0.280
	FRAP	*R* ^2^ = 0.657, *P*=0.020	*R* ^2^ = 0.705, *P*=0.010
	TAC	*R* ^2^ = 0.383, *P*=0.219	*R* ^2^ = 0.554, *P*=0.062
EE	DPPH	*R* ^2^ = 0.412, *P*=0.184	*R* ^2^ = 0.355, *P*=0.257
	FRAP	*R* ^2^ = 0.289, *P*=0.363	*R* ^2^ = 0.419, *P*=0.175
	TAC	*R* ^2^ = 0.494, *P*=0.102	*R* ^2^ = 0.245, *P*=0.443

AE, aqueous extract; HE, hydroethanolic extract; EE, ethanolic extract; DPPH,2, 2-diphenyl-1 picrylhydrazyl; FRAP, ferric reducing antioxidant power; TAC, total antioxidant capacity.

## Data Availability

The data used to support the findings of this study are available from the corresponding author upon request.
